# Toward a comprehensive profiling of alternative splicing proteoform structures, interactions and functions

**DOI:** 10.1016/j.sbi.2024.102979

**Published:** 2025-01-07

**Authors:** Elodie Laine, Maria Inés Freiberger

**Affiliations:** 1 Sorbonne Université, CNRS, IBPS, Laboratory of Computational and Quantitative Biology (LCQB), UMR 7238, 75005 Paris, France; 2 Institut universitaire de France (IUF)

## Abstract

The mRNA splicing machinery has been estimated to generate 100,000 known protein-coding transcripts for 20,000 human genes (Ensembl, Sept. 2024). However, this set is expanding with the massive and rapidly growing data coming from high-throughput technologies, particularly single-cell and long-read sequencing. Yet, the implications of splicing complexity at the protein level remain largely uncharted. In this review, we describe the current advances toward systematically assessing the contribution of alternative splicing to proteome function diversification. We discuss the potential and challenges of using artificial intelligence-based techniques in identifying alternative splicing proteoforms and characterising their structures, interactions, and functions.

## Alternative splicing and proteome diversity

**Figure 1:**
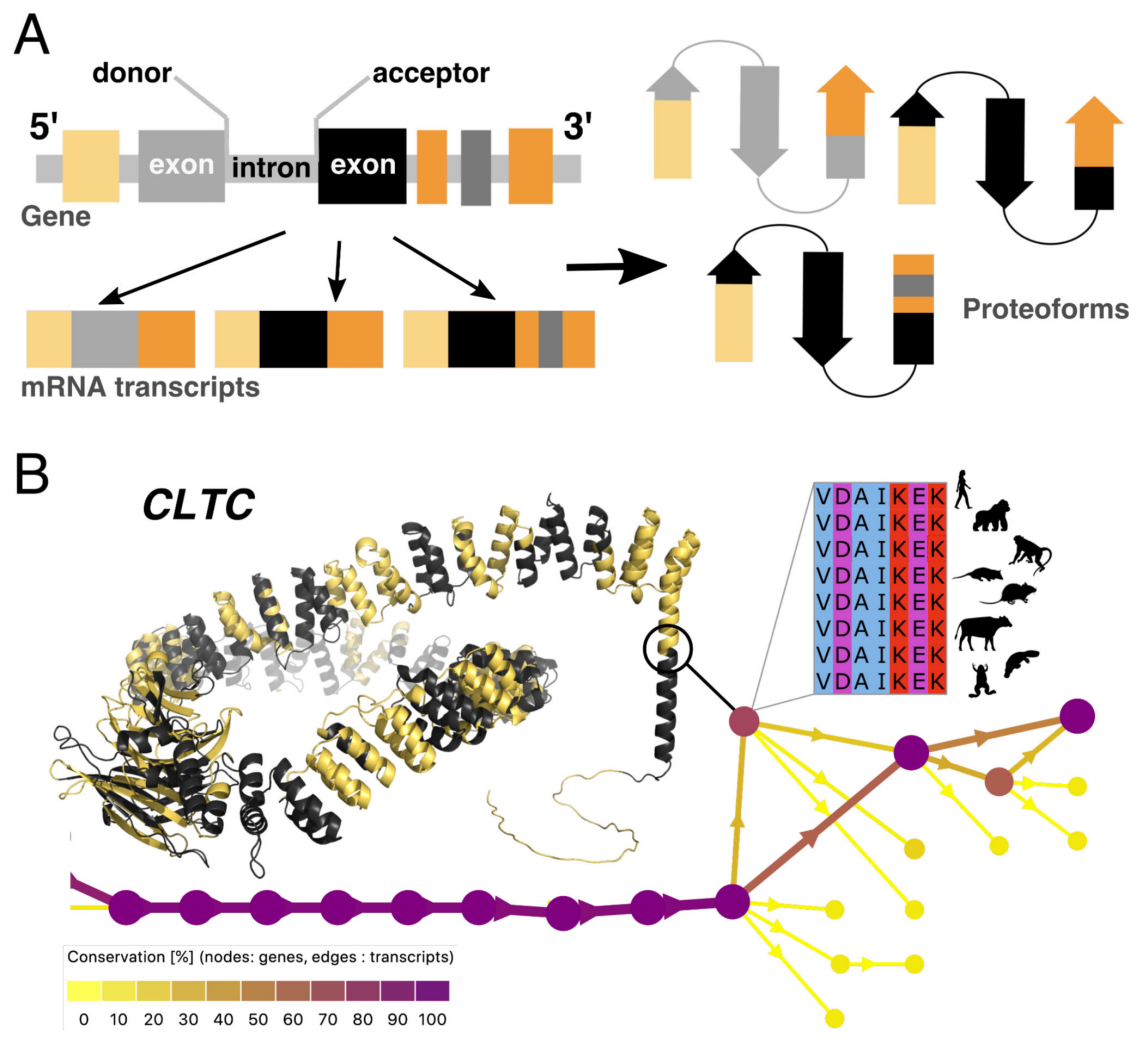
Basics of alternative splicing and an illustrative example. **(A)** Schematic representation of a eukaryotic gene, focusing on the protein-coding region. The high boxes are the exons, separated by thin boxes depicting the introns. The donor and acceptor splice sites are at the exon-intron and intron-exon boundaries. Three mRNA transcripts corresponding to different combinations of exons are shown. They may be translated into proteoforms adopting different shapes (arrows: β-sheet, rectangle: α-helix). **(B)** The human gene *CLTC* encodes the 1675-residue long clathrin heavy chain 1 whose 3D model (https://alphafold.ebi.ac.uk/search/text/Q00610 [26]) is displayed as cartoons. The evolutionary splicing graph at the bottom recapitulates the alternative proteoforms observed over eleven species from human to zebrafish (http://www.lcqb.upmc.fr/Ases http://www.lcqb.upmc.fr/Ases/results?jobid=KXFyXXbHm3 [27]), focusing on the C-terminal protein region. The nodes or s-exons are coloured according to their conservation level (species fraction) on the graph and delineated in black and yellow on the 3D structure. The most conserved event, present in human, gorilla, macaque, rat, cow, opossum, platypus, and frog, is a 7-aa insertion in the protein C-terminal trimerization domain. This insertion, supported by transcriptomic and proteomic data, triggers a switch from spherical clathrin-coated pits to flat clathrin lattices during muscle cell differentiation [9].

The recent advances in high-throughput sequencing, imaging, and proteomics have revealed an incredible complexity behind the classical protein sequence-structure-function paradigm [1]. In particular, in multicellular organisms, alternative splicing (AS), together with alternative promoter usage and alternative polyadenylation, can produce multiple mature messenger RNAs, or *transcripts*, from a single gene [2] (Figure 1A). Some of these transcripts will lead to different protein isoforms, or *proteoforms* [3], that may adopt 3D structures with different shapes [4], interact with distinct cellular partners [5], and perform divergent or specialised functions [6,7]. For example, a 10-amino acid (aa) substitution between two proteoforms of the protein kinase JNK1 changes its binding partner preferences, thus triggering different stress responses [8]. Similarly, while a shorter clathrin proteoform self-assembles into spherical coats in neurons, a 7-aa longer one forms flat plaques in muscle cells [9] (Figure 1B). The plethora of scenarios in which AS modulates protein functions and interactions [10] play essential roles in muscle fibre diversification [11], nervous system development [12], and innate immunity [13]. Moreover, the combinatorial expression of various proteoforms can influence disease susceptibility [14] and signalling outcomes in response to drugs [15], and AS misregulation is often linked to various diseases, including cancer [16,17].

Experimentally determining how much of the splicing complexity uncovered by RNA-seq [18] contributes to protein diversity remains a long-standing challenge [19]. Higher AS rates are typically observed for species with lower effective population sizes, suggesting that they result from genetic drift of the splicing machinery [20]. Along this line, analysing mass spectrometry data on a large scale initially suggested that most highly expressed human genes have only one dominant proteoform [21]. However, improved analysis protocols using custom peptide databases or integrating long-read transcriptomics reported many more proteoforms [22–23]. Furthermore, a major fraction of alternative transcripts are engaged by ribosomes [24] and a recent deep-coverage mass spectrometry study revealed evidence that most frame-preserving alternative transcripts are translated [25].

**Figure 2:**
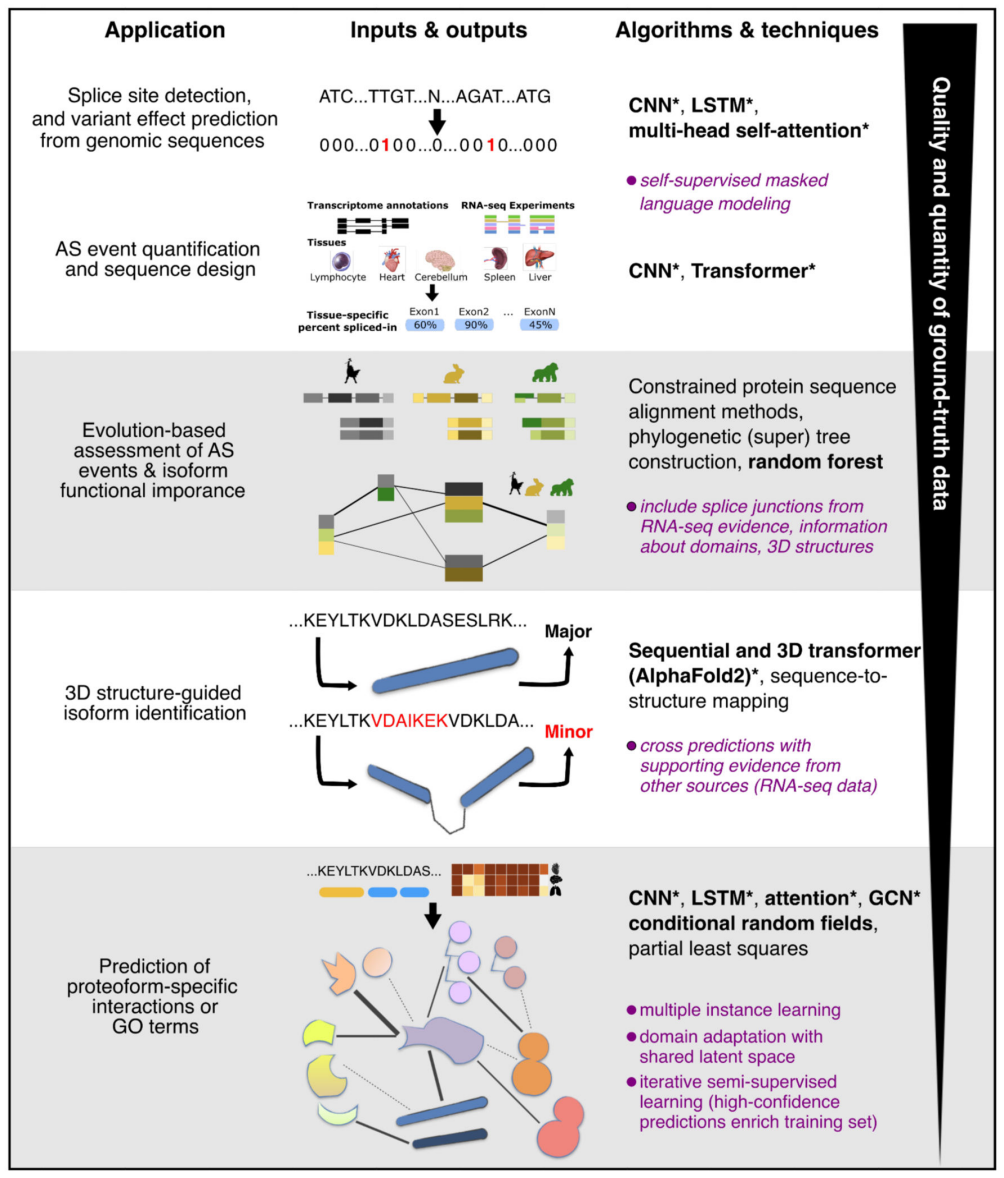
Overview of methods and applications for shedding light on alternative splicing contribution to proteome diversification. We mostly focus on approaches developed in recent years, which often integrate one or more of the mentioned algorithms and techniques. The latter are classified into heuristic (plain text), classical machine learning (bold) and deep learning (starred bold). The bullet points in italics and purple indicate strategies for coping with lack of ground-truth data.

Emerging high-throughput computational methods efficiently leveraging large amounts of protein-related data represent an opportunity for complementing experimental evidence, toward refining the definition of gene structures, quantifying the alternative usage of exons, and improving our understanding of AS impact on protein 3D structures, interactions, and functions (Figure 2).

## Deciphering the splicing code

Computationally recognising the genomic signals determining which protein-coding segments will be spliced together by the spliceosome is a fundamental step for describing proteoform diversity. Donor (or 5’) splice sites, at the exon–intron junctions, typically feature a GT dinucleotide, and acceptor (or 3’) splice sites, at the intron–exon junctions, an AG dinucleotide (Figure 1A). Nevertheless, not all GT-AG pairs signify splicing, some splice sites may feature non-canonical patterns, and other environmental factors and regulatory signals come into play, making the task challenging [28]. While early splicing code models relied on putative regulatory features [29], the most recent predictors recognize splice sites directly from raw genomic or pre-mRNA nucleotide sequences [30–37] (Figure 2). They borrow deep learning architectures from image classification like convolutional neural networks (CNN) or from natural language processing like transformers.

Among these next-generation splicing predictors, the ultra-deep residual CNN-based model SpliceAI [30] analyses pre-mRNA genomic sequences to compute the probability of each residue being a splice donor, splice acceptor, or neither. It has proven effective in predicting splicing alterations, exon skipping, and splicing rescue through cryptic site activation [38]. SpliceAI performance is matched by large language models (LLMs) pre-trained to reconstruct masked or corrupted genomic sequences at scale [35]. SpliceAI and LLMs evaluate thousands of nucleotides around the position of interest, up to 32 kb with HyenaDNA [39]. However, accounting for wider contexts does not necessarily translate into improved accuracy [35] and may not reflect what the spliceosome can recognize in the cell [31–32]. Several predictors reach state-of-the-art accuracy by focusing on shorter sequences, and a few further constrain their architectures to more closely mimic the splicing process and improve interpretability [32,37]. For instance, the SAM splice site predictor is explicitly informed with knowledge about sparse RNA-binding protein motifs [37].

Models trained on one species typically exhibit low generalisation capability to other species [32]. Scalzitti and co-authors explicitly addressed this issue by training the Spliceator model [31] on a carefully curated benchmark set encompassing a hundred phylogenetically diverse organisms. In addition, using the predictors to assess the impact of alterations in the input sequence on the splicing outcome requires choosing appropriate thresholds that may depend on factors not explicitly modelled, such as splice site strength or exons’ baseline inclusion rates [40]. Pangolin [33] and TrASPr [36] make a step forward by quantifying AS splice site usage and events under specific conditions (*e.g*., tissue), opening the way to design genomic sequences tuned to desired splicing outcomes (Figure 2).

## Leveraging evolutionary conservation

Evolutionary conservation often serves as a reliable indicator of function, suggesting that natural variations induced by AS, and selected through evolution, likely fulfil important functional roles under physical and environmental constraints. For instance, mutually exclusive tandem duplicated exons (MXE) are an example of ancient AS events that have critical functional significance [41,42]. Substitutions in these exons have likely contributed to tissue and organ evolution in metazoans and have clinical implications in humans [41]. More broadly, cross-species conservation is the most discriminating feature for state-of-the-art prediction of transcript biological relevance at the protein level [43]. Reciprocally, AS variations disrupting conserved active sites and functional domains are unlikely to result in functional translated products.

To accurately assess the evolutionary conservation of AS events, it is necessary to match exons, splice junctions, or transcripts/proteoforms across species. Early methods have relied on genomic sequence alignments to identify orthologous exons [44]. However, difficulties arise from large indels, ambiguities between highly similar or short sequences, or lack of plausible matches for highly divergent sequences. A few recent methods address these challenges by adopting an end-product perspective, working with the amino acid sequences of the putative proteoforms enriched with knowledge about the gene structure [45,46].

In particular, evolutionary splicing graphs provide a compact representation summarising the full proteoform diversity observed for a set of orthologous genes [45] (Figure 1B). By extending the concept of splicing graphs [47] to several species, they allow for identifying (sub-)exon orthogroups (nodes), quantifying splice junction usage (edges), and investigating exon co-occurrence (paths). Building such graphs from annotations and RNA-seq data across a dozen species spanning 800 million years of evolution showed a clear link between conservation, tissue regulation, and functional relevance of alternative transcripts [45]. Furthermore, disentangling orthologous from paralogous relationships between entire transcripts/proteoforms [48,49] and simulating or reconstructing transcript phylogenies [8,50] can help to infer evolutionary scenarios explaining AS-induced protein function diversification.

## Modelling proteoform 3D structures

While only a few tens of alternative splicing proteoforms have experimentally resolved 3D structures, the advent of high-throughput deep learning-based protein structure prediction methods, which achieve near-experimental accuracy, has enabled systematic probing of AS impact on protein folds and structural stability [51,52]. Sommer and colleagues [52] proposed using AlphaFold2 [26] average predicted local distance difference test (pLDDT) score as a measure of "biological functionality" for genome annotation (Figure 2). One should be cautious with such an approach because short, well-folded fragments from larger proteins often display higher pLDDT scores than the full-length protein, potentially misleading functional interpretations. Reciprocally, a proteoform with a longer inter-domain disordered linker would be penalised in terms of pLDDT while it may acquire the ability to translocate to another cellular compartment or bind to new partners. The authors partially addressed these issues by applying a series of filtering criteria based on proteoform length, pLDDT distribution and RNA-seq expression data [52].

**Figure 3:**
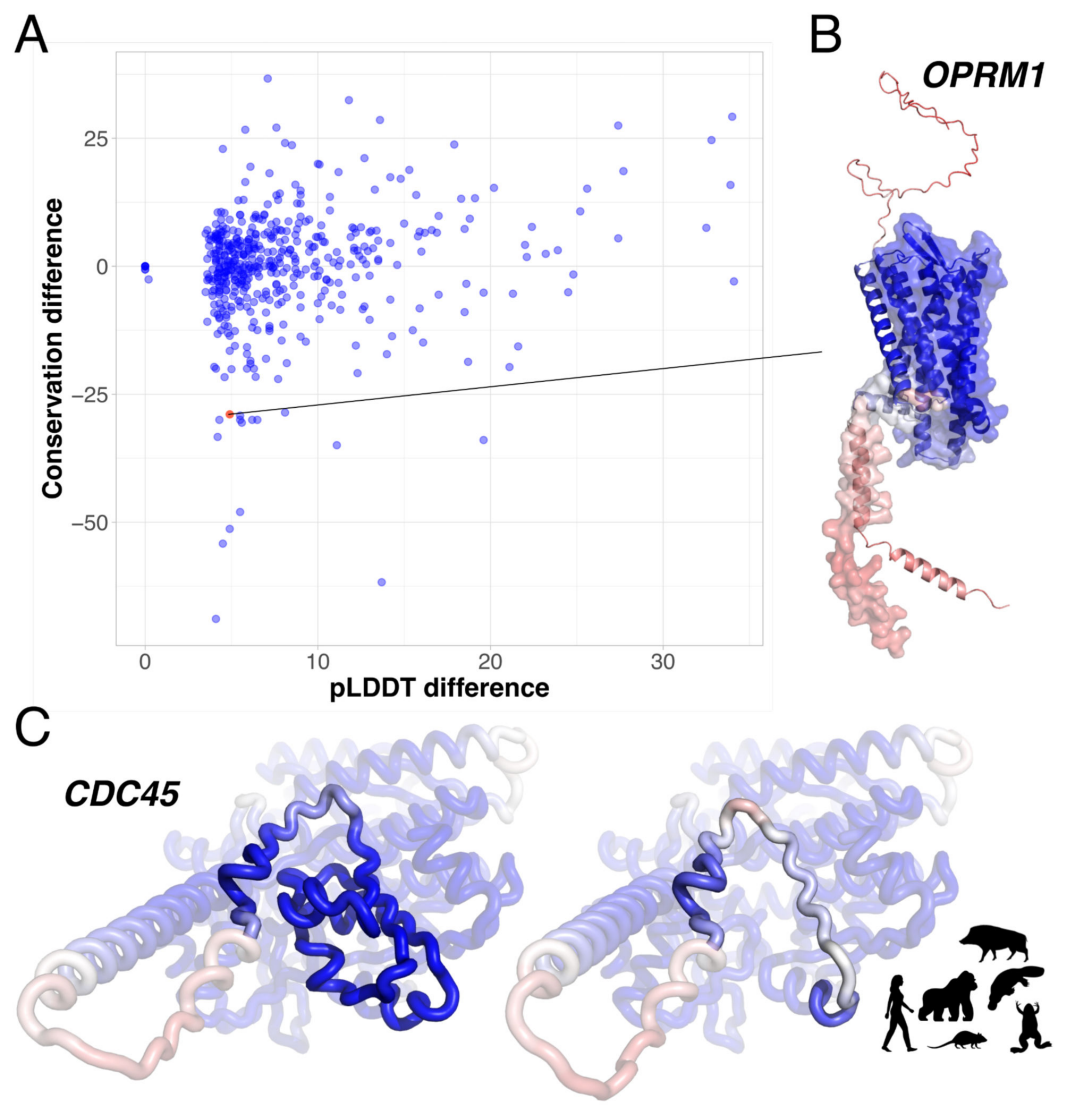
Structural modelling and evolutionary conservation of alternative splicing events. **(A)** The alternative proteoforms identified in [52] as having more stable structures (higher pLDDT, x-axis) than MANE-annotated primary proteoforms are not necessarily more conserved in evolution (y-axis). ThorAxe estimated evolutionary conservation as the average splice junction usage across a dozen species, from human to nematode (averaged transcript fraction mean in [45]). **(B-C)** AlphaFold2-predicted 3D models (from isoform.io v3.1 [52]), coloured according to the pLDDT, from red (low) to blue (high), for OPRM1 and CDC45 proteoforms. **(B)** The mu opioid receptor OPRM1 MANE proteoform (in cartoon) represents a full-length canonical G protein-coupled receptor (GPCR) structure with an extracellular N-terminus followed by seven transmembrane alpha-helices. The candidate alternative proteoform (in surface) lacking the N-terminus and the first transmembrane helix has a higher pLDDT but much lower conservation. This truncated form does not retain the function of the full-length receptor, while it could modulate the function of other GPCRs [61]. **(C)** AlphaFold2 modelled exon 4 skipping in CDC45 as cut-and-stitch. The exon seems essential for function since it encodes a part of the RecJ nuclease-orthologue’s DHH domain. Yet, exon 3-5 junction is expressed in low levels in several tissues based on GTEx mRNA data [62], and ThorAxe detected it in several species, from human to frog, based on Ensembl annotations (https://www.ensembl.org/).

They identified 940 alternative human proteoforms with pLDDT scores suggesting they might be more functionally active than those annotated as primary in the MANE (Matched Annotation from NCBI and EMBL-EBI) database [53]. Evolutionary wise, these alternative proteoforms span a wide range of conservation levels (Figure 3A). Some of them, like the mu opioid receptor OPRM1 proteoform lacking the N-terminus and first helix, are much less conserved than their MANE counterpart (Figure 3A-B). These observations suggest that cross-species conservation could be useful to refine the approach.

In addition, the suitability of AlphaFold2 for predicting some alternative proteoform structures is questionable. For instance, AlphaFold2 tends to model AS-induced large deletions in well-folded domains through cut-and-stitch with low confidence scores assigned to the stitched region (Figure 3C). A recent study highlighted how this comparative modelling-like behaviour produces physically unrealistic 3D models for alternative proteoforms where patches of hydrophobic residues are exposed to the solvent [54]. This limitation, also shared by protein Language Model(pLM)-based protein structure predictors, emphasises the need for methods tailored to extract signals from AS-induced sequence variations.

## Variations on the same theme

AS of duplicated protein regions enables fine-tuning of protein function without altering the protein fold [42,55]. Systematically mapping the MXEs identified in 5 high-quality Metazoan genomes to the CATH-derived protein domain functional families (FunFams) revealed that MXE-specific residues are mostly located on the protein surface and cluster at or near protein functional sites [42]. A parallel study focusing on human and relaxing the criterion of mutual exclusivity confirmed these findings on AlphaFold-predicted 3D models and further showed that MXE amino acid substitutions tend to affect disordered residues or residues that do not directly bind ligands [55].

Beyond MXE pairs, the combinatorics of AS-induced topological rearrangements of similar exonic sequences can be more complex [56]. For instance, AS produces four different combinations of calmodulin-binding motifs in myosin 1b's lever arm [45], thereby modulating the protein’s ability to sense mechanical forces and hence to pull membranes [57]. The giant skeletal muscle protein nebulin gives a more extreme example where over 100 protein regions, each corresponding to one or more ~35 aa-long nebulin-like motifs, are subject to 47 inclusion/exclusion events across a dozen species, including two primates, six other mammals, zebrafish and frog [56]. In the leucine-rich-repeat containing G-protein coupled receptor 5, the number of repeats is modulated by three inclusion/exclusion AS events, among which two are conserved from human to frog. When the repeats have well-defined structures, the exon-intron gene structure and the AS-induced variations tend to preserve their integrity and can help refine their boundaries [58].

Jointly analysing the proteoforms of tandem repeat-containing proteins observed in several species can help to gain insights into how these protein regions have evolved essential functions [59] and more broadly, into the relationship between AS and gene duplication [60].

## Unveiling AS impact on interactions and functions

AS events can rewire protein-protein interaction (PPI) networks by altering functional motifs in intrinsically disordered regions, gaining or losing entire structured domains, or inducing small changes in their interacting surfaces [5,6,10,44,63–64]. To move forward in assessing the functional role of AS on a system biology level, researchers have systematically mapped known human protein interactions and functional annotations on genomic exons [65–67]. The Domain Interaction Graph Guided ExploreR (DIGGER) database even enables an exon-centric exploration of human PPIs by exploiting information about physical contacts between residues from experimental 3D complexes [65]. Nevertheless, experimental data about exon-exon interactions cover only about 5% of all known human PPIs, stressing the need for producing and analysing high-quality 3D models [68].

Machine learning approaches have emerged to predict proteoform-specific interactions and functions, but they face challenges such as the scarcity of ground-truth data and the heterogeneity of proteoform-related data [69] (Figure 2). Multiple instance learning (MIL) algorithms address the first issue by integrating genomic and protein-level information. In these frameworks, a gene is conceptualised as a *bag* containing its proteoforms, which are treated as *instances* within the bag. Functional annotations are initially assigned at the gene level (the bag) and then propagated to its proteoforms (the instances), with refinements made to ensure proteoform-specific accuracy [70–72]. The attention mechanism can be used to increase the difference between isoform pairs from the same gene bag in the context of interaction prediction [71] or to account for the fact that two or more proteoforms from the same gene can work together to accomplish the same function [73]. Semi-supervised learning offers an alternative approach to MIL in which high-confidence predictions generated from unlabeled samples are iteratively added to the training set to refine the model [74].

In recent years, modular deep learning architectures have been proposed to deal with the second issue, the heterogeneity of the input data [69,75]. They typically consider nucleotide and/or aa sequences, RNA-seq data and optionally domain composition or domain-domain interactions. For instance, the proteoform function predictor DIFFUSE combines features extracted with convolutions from proteoform aa sequences, transcript co-expression RNA-seq data aggregated with probabilistic graphical models, and information about evolutionary conserved domains treated with LSTM [69]. Further developments aim at a more unified integration, either through end-to-end deep learning architectures [73] or by projecting the input data into a common latent space with partial least squares [76].

Beyond protein-protein complexes, AS events affecting protein interactions with nucleic acids, lipids, carbohydrates and small molecules have also been documented [10]. Protein-ligand interactions established through binding interfaces found in specific proteoforms expressed in specific tissues provide new opportunities for drug design and targeting strategies, as exemplified by the GPCR superfamily [15].

## Concluding remarks and future outlook

Emerging deep learning paradigms show promise in clarifying how AS contributes to protein functional diversification. Unlike traditional methods, and provided sufficient compute and data, deep learning techniques excel at automatically extracting meaningful features directly from raw data, eliminating the need for labour-intensive feature engineering. However, a lack of high-quality ground-truth data poses significant challenges for training and evaluating machine learning, especially deep learning approaches. Gene predictions frequently contain errors [77], and alternative splicing proteoform-specific functional annotations and interactions are scarce, partial, and likely noisy [74]. Defining reliable negative training sets is extremely difficult because it is almost impossible to demonstrate that splice variants do not have any function or cannot interact with one another [43]. Traditional train/test splitting may lead to training sets that are not representative enough. Data augmentation is often impractical because we do not know or control the impact of small changes on protein interactions and functions.

Self-supervised representation learning could allow for overcoming some of these limitations. Large foundation models, in particular, enable the transfer of knowledge across genes, proteins, and species by leveraging universal representation spaces. This approach enhances robustness and reduces sensitivity to errors and ambiguities in input data preprocessing, such as those arising from multiple sequence alignments. However, not all learned representations may achieve high quality due to uneven coverage and biases in the training data. Looking ahead, multimodal generative models like ESM-3 (https://github.com/evolutionaryscale/esm), which build upon the breakthroughs in protein structure prediction, are starting to jointly reason over protein sequences, structures, and functions. These models open exciting possibilities for finely capturing proteoform differences and even designing or tweaking specific proteoforms.

Exciting progress is also being made in top-down proteomics [78] and nanopore protein sequencing [79] aided by machine learning for signal processing and recognition. They let us envision the possibility of comprehensively identifying proteoforms generated by AS and post-translational modifications. Beyond identification, emerging RNA-targeting strategies for programmable manipulation of AS – such as synthetic splicing factors or recruitment of the endogenous splicing machinery – open the way to systematic functional exon screening [80]. Although challenges remain before achieving high resolution and throughput, these experimental techniques are starting to peel back the layers of complexities of protein states and functioning in the cell.
